# Up to date concepts about Von Willebrand disease and the diagnose of this hemostatic disorder

**Published:** 2014-09-25

**Authors:** I Buga-Corbu, C Arion

**Affiliations:** * "Carol Davila" University of Medicine and Pharmacy, Bucharest; ** "I. C. Fundeni" Pediatrics Clinic, "Carol Davila" University of Medicine and Pharmacy, Bucharest

**Keywords:** hemostase, hemostatic disorders, diagnostic approach

## Abstract

Abstract

The authors review the current data in literature regarding the recent knowledge about hemostase, coagulation and clinical and laboratory diagnostic algorithms of hemostatic disorders. They also present the pathological classification of bleeding disorders - the basis to clinical approach of these diseases.

Abbreviations: AD=autosomal dominant; Ag=antigen; DNA=deoxyribonucleic acid; ADAMTS13=serum metalloproteinase; AR=autosomal recessive; Arg=arginine; RNA=ribonucleic acid; VWD=von Willebrand disease; Cys=cysteine; C1—C9=factors of the seric complement; ELISA=enzyme linked immuno assay; FI---FXIII=plasmatic factors of coagulation; Glu=glutamines; Pg=platelet glycoprotein; HMW=high molecular weight; IL=interleukin; SLE=systemic lupus erythematosus; Met=methionine; PFA=automated study test of platelets aggregation; RCo=ristocetin cofactor; RI PA=ristocetin induced platelet aggregation; Tyr=tyrosine; VWF= von Willebrand factor

 Physiological hemostasis 

 The hemostasis represents the ensemble of phenomena that start together with the production of a vascular gap, having as an outcome the obstruction of the gap and the stopping of the hemorrhage. 

 Two stages of the hemostasis are distinguished (theoretically, in reality the two stages take place concomitantly): primary hemostasis (vascular-platelet time) and secondary hemostasis (coagulation).

 Primary hemostasis 

 The vascular lesion triggers the vasoconstriction in the affected area by short, axon reflexes. The vasoconstriction is maintained by the action of some substances such as noradrenalin and adrenalin, which are released locally, at the level of the sympathetic nerve endings or which have got there via blood. The vasoconstriction determines a decrease of the circulation in the affected area and diminishes the hemorrhage. 

 The platelets intervention in this stage is complex and contains more stages, which are theoretically distinct: 

 Platelet adhesion 

 The disruption of the endothelial barrier produced by the vascular lesion leads to the exposure of the endothelial collagen; through von Willebrand factor (VWF), which is an adhesive synthesized glycoprotein, multimerized and stored in specific cellular granules, in endothelial cells and platelets, being present in the plasma; together with the GPIb specific platelet receptor (as well as some other platelet receptors for collagen: GP VI, α2β), the circulant platelets adhering to the subendothelium. 

 The activation of the platelets and the release of the factors that were stored in the platelets granules 

 Concomitantly with the adhesion, a complex series of metabolic reactions, which define the platelets activation and the release of some factors that were stored in the platelets granules, with important roles in hemostasis: ADP, serotonin, thromboxane A2, fibronectin, TPA, factor V, etc., takes place. 

 Moreover, a morphological transformation of the platelets takes place – the transformation from the flat disk in circulation with pseudopods. 

 Platelet aggregation 

 The circulating platelets agglomerate at the level of the vascular gap, the connection between them being made through the fibrinogen and the specific platelet receptor GPII b/IIIa. 

 Secondary hemostasis (coagulation)

 It represents a set of chemical reactions initiated by the expression of the tissue factor (TF) at the level of the endothelial lesions (on the local fibroblasts) which imply specific plasmatic proteins. 

 − the coagulation factors, which activate "in cascade" and lead to the formation of the permanent hemostatic plug (fibrinoplatelet).

 Currently, the following stages, which develop concomitantly in real time (**Fig. 1**) can be distinguished in this complex process:

**Fig. 1:**
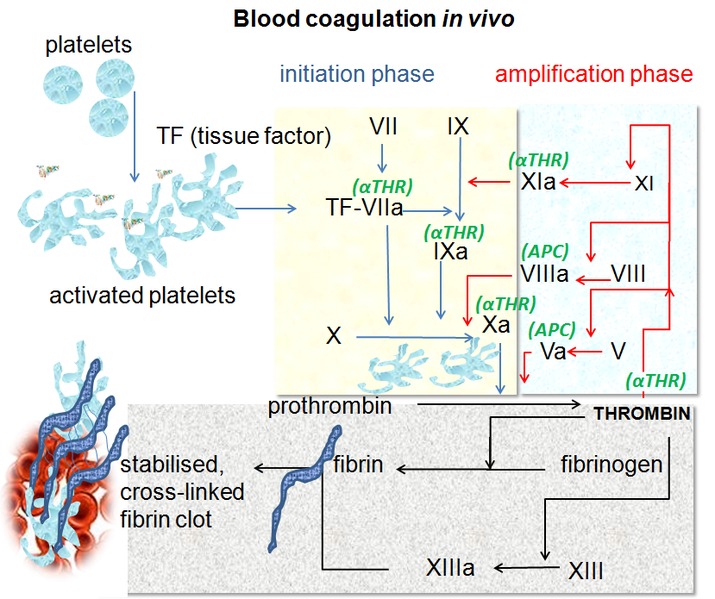
The coagulation in real time [7]

The initiation of the coagulation cascade 

 The TF cellular expression in the place of the endothelial lesion leads to the activation of F.VII. The activated FVII (F.VIIa), leads to the activation of F.X and F.IX. F.Xa acts on F.V., activating F.Va enzymatically activates F.II (prothrombin) in thrombin. 

 This stage implies the extrinsic system of coagulation. 

 The little quantities of thrombin produced in the initiation stage have important effects on the subsequent development of hemostasis: 

 - Activate the platelets 

 - Accelerate the platelet aggregation 

 - Activate important plasmatic factors of coagulation (the extrinsic system of coagulation) – F.VIII , F.V, F.XI .

The amplification 

 Takes place on the surface of the platelets through the formation of some enzymatic complexes that also contain the platelet phospholipid. 

 F.XIa acts on F.IX which is enzimatically activated. F.VIII is activated by the little quantities of thrombin produced in the previous stage, (FVIIIa), together with FIXa and the platelet phospholipid assembles on the surface of the platelets, the enzymatic complex being called tenase. 

The tenase acts on F.X, which is activated by (F.Xa). F.Xa together with F.V.a, (also activated by the little quantities of thrombin produced in the previous stage) and with the platelet phospholipid assembles on the surface of the enzymatic platelets – prothrombinase. The prothrombinase acts on prothrombin transforming it into an active thrombin; in this stage, considerable quantities of thrombin are generated. 

The propagation 

 Consists in the amplification of the phenomena described in the previous stage and is also produced on the surface of the platelets. 

F.Xa included in the complex of prothrombinase is protected by the inactivation exerted by the TF path inhibitor factor, which allows the accelerated development of the thrombin production. 

The fibrin formation 

 The thrombin acts on fibrinogen (F.I), a plasmatic protein in considerable concentration: 200-400 mg/dL. The thrombin action leads to fibrinopeptides A and B splitting and to the aggregation of residual polypeptide chains, with the formation of fibrin monomers. They polymerize (fibrin polymers), which are disposed in a tridimensional network, that also contains figurative elements of the blood. 

The stabilization of the clot 

 F.XIII (FSF, the fibrin stabilization factor) is also activated by the thrombin, leading to the formation of a fibrin stable clot. Thrombin also favors the aggregation of the platelets, adding a dense platelets aggregate to this fibrin clot. 

The clot retraction (thrombodynamic stage) 

 In this stage, the platelets included in the clot actively intervene through their contractile mechanisms, leading to the expulsion of the serum from the meshes of the fibrin network; this way a dense platelet aggregation is formed, connected to strips of fibrin, becoming the definitive hemostatic stop, which obstructs the vascular gap. 

The classification of hemorrhagic syndromes 

 The disruption of one or more stages of hemostasis leads to the appearance of hemorrhagic syndromes. From an etiopathogenic point of view, these can be due to the vascular anomalies, the platelets anomalies – quantitative or qualitative – coagulopathy or hyperfibrinolysis. 

 Each of the anomalies of the hemostasis stages can be congenital (hereditary, familial) or acquired. A presentation of these hemorrhagic syndromes is presented in Table 1.

Anomalies of the first stage of hemostasis 

1. Vascular anomalies 

1.1. Vasculitis 

1.1.1. Infectious

- Bacterial endocarditis 

- Meningococcemia

- Rickettsioses

- Enteroviruses infection 

 1.1.2. Autoimmune

 - SLE 

 - DM/PM

 - SD/SS

 - JIA

 1.1.3. Vascular dystrophies 

 - Hereditary anomalies of the blood vessels and of Ehlers-Danlus Syndrome conjunctive tissue, Pseudoxanthoma elasticum, Marfan Syndrome, Imperfect osteogenesis, Hereditary hemorrhagic telangiectasis, etc.

 - Earned = scurvy, severe MPE 

 1.1.4 Purpura / post-traumatic bruises 

 1.1.5 Factitive purpura 

 2. Platelets anomalies 

 2.1.Quantitative (thrombocytopenia) 

 2.1.1. Hereditary thrombocytopenia 

 - TAR syndrome

 - Amegakaryocytic thrombocytopenia associated with radio/ulnar synostosis/ associated with the agenesis of the corpus callosum. 

 - Shwachman syndrome 

 - Fanconi anemia

 - Wiskott-Aldrich syndrome

 - From the metabolic hereditary diseases (methylmalonic acidemia, ketotic glycinemia, isovaleric acidemia, ketotic hyperglycinemia, etc.).

 - From the medullary infiltrative diseases (congenital osteoporosis, tesmoised diseases - MPZ)

 2.1.2. Earned thrombocytopenia 

 - Immune/autoimmune (PTI, AHAI, ALPS, inflammatory diseases of the conjunctive tissue/ drug-induced – AINS, Vancomycin Chinin, Quinidine, etc.)

 - Allo/isoimmune (neuritis, post-transfusion, GVHD, the rejection of the solid organs graft).

 - Non-immune 

 -Infectious: congenital rubella, HIV, VEB, CMV, other herpes-viruses, hepatic viruses, parvovirus, B19, rougeole, toxoplasmosis, tuberculosis, malaria, ehrlichiosis, etc. 

 -Microangiopathy: Kassabach syndrome

 -Merrit, CID, SHU, PTT, TCSH microangiopathy 

 -Drug specific: heparin, chloramphenicol, phenicol, tuberculostatic, AINS, cytostatic, Rx. ionisant, diuretic, protamine sulphate, etc. 

 -By raised splenic seizure (congestive hypersplenism – HTP, infiltrative splenomegaly, secondary to constitutional hemolytic anemias, Felty syndrome, etc.). 

 -Medullary infiltration from malignities (LAL, LAM, LGC, lymphomas, tumor metastases, langerhans and malignant histiocytosis).

 -SAA

 2.2. Qualitative (thrombocytopathia) 

 2.2.1. Congenital (hereditary)

 - Deficits of thrombocyte adhesion (Bernard – Soulier syndrome)

 - Deficits of thrombocyte aggregation (Glanzmann Thrombasthenia) 

 - Deficits of synthesis and release of the factors stocked in the platelets granules (the grey platelet syndrome, Hermansky-Pudlak syndrome, Chediak-Higashi syndrome, May-Hegglin anomaly, anomalies of the path of arachidonic acid, deficit in the calcium mobilization).

 2.2.2. Earned 

 - Type I glycogenosis 

 - Drug-induced (Aspirin, etc., AINS, Cephalosporin, Carbenicillin, Dextran, Valproic acid, phosphodiesterase inhibitors) 

 - Myeloprolipherative syndromes 

 - Uremia

 - Liver failure

 - Amyloidosis

 - Induced by autoantibodies 

 II. Anomalies of coagulation (coagulopathy) 

 1. Hereditary (congenital) 

 1.1. Isolated deficits of some factors of coagulation 

 1.1.1. Factor VIII (hemophilia A) deficit 

 1.1.2. Factor IX (hemophilia B) deficit 

 1.1.3. Factor XI (hemophilia C) deficit 

1.1.4. Factor X deficit 

 1.1.5. Factor VII (Alexander) deficit 

 1.1.6. Factor V (Owren parahemophilia) deficit 

 1.1.7. Factor II congenital deficit 

 1.1.8. AI congenital hypofibrinogenemia 

 1.1.9. Factor XIII – FSF deficit 

 1.1.10. Von Willebrand Disease

 1.2. Hereditary anomalies which interest more than one factor of coagulation (ex. Associated deficit of f.VIII and f.V) 

 2. Earned (acquired) 

 2.1. Vitamin K deficit 

 2.2. Consumption coagulopathy (CID secondary) 

 2.3. Production coagulopathy (secondary to hepatic insufficiency) 

 2.4. Overdose/ intoxication with anticonvulsive drugs 

 (heparin, cumarinic)

 2.5. Snake venom 

 2.6. Earned Hemophilia A 

 2.7. Earned Von Willebrand syndrome

2.8. Congenital cardiac diseases 

 2.9. Extracorporeal circulation 

 III Hyperfibrinolithic syndromes 

 1.Congenital 

 1.1. Alpha2 – antiplasmin deficit 

 2.Earned

The diagnosis of hemorrhagic syndromes 

 Clinical examination 

 The diagnostic approach of hemorrhagic syndromes starts with the clinical evaluation of the child. The first differentiation assigns the bleedings to a local cause or a systemic affection. From this point of view, it is important to find out the answer to the previous requests of hemostasis (the ligature and the sectioning of the umbilical cord, detachment of the umbilical blunt, dental eruption, dental extractions, surgeries, menarche, etc.). 

 The usual bleeding area also gives the diagnosis guiding elements: 

 - Cutaneo mucous hemorrhages (bruises, purpura, epistaxis, gums hemorrhages, menorrhagia, etc.) are especially met in the anomalies of primary hemostasis (thrombocytopenia, thrombocytopathia, von Willerbrand disease, etc.). 

 - "Spontaneous" hemarthrosis and profound hematomas of the soft tissues are characteristic to coagulopathies. 

 The timing of the appearance of hemorrhages according to the shutter traumatism represents a differentiation factor: bleedings with immediate post-traumatic start are characteristic for vasculo-platelet anomalies, while the hemorrhages with a late start compared to the moment of the trauma are met in coagulopathies. 

 The answer of the hemorrhages to the usual hemostatic measures (tamponade, suture of the wounds, vessels ligature, etc.) is also different in the anomalies of primary hemostasis – in which we obtain a clinically satisfactory answer compared to the coagulopathies in which only the substitution of the deficient factors of coagulation leads to the control of hemorrhages. 

 The drugs administered to the patient in the past and in the present can affect the hemostasis. 

 A last aspect which can favors important information for the diagnosis is the familial history of hemorrhagic diathesis; this way we can answer the question whether the hemorrhagic syndrome is constitutional (hereditary, familial) or acquired. 

 The laboratory screening of hemostasis 

 The examination of the patient continues with the hemostasis laboratory screening investigations, which will allow the objective placement of the patient in one of the major category of hemorrhagic syndromes or which will allow even the diagnosis of the affection in some cases. 

The number of platelets in S.P. (N: 150 – 400.000/ml)

 The prothrombin time (T.Quick, PT ; N: 11,8 – 14,0 sec.)

 The activated thromboplastin time (a TPA; N = 23,8 – 32,0 sec)

 The plasma fibrinogen (gravimetric or immunologic method; N =(00 200– 400 mg/ dL).

Although at present there are various laboratory tests for the investigation of hemostasis, there is no consensus regarding the tests that are unanimously used in the screening of hemorrhagic syndromes, especially pre-operatory. 

 Table 2 offers an inventory of the hemostasis screening tests that are used most often. 

The differential diagnosis compared to the results of the hemostasis screening tests is presented in Table 3. 

APTT ↑

 ↓ / PT = N

 - Factors VIII, IX and XI deficits (The factors XII, PK/K, HMWK deficits, just like the lupus antigoagulant can prolonge APTT, but are not tipically associated with the hemorrhagic diathesis). 

 - Inhibitors of factors VII, IX and XI (The differential diagnosis between the deficit of the coagulation factors and the presence of the inhibitors of these factors in the plasma is done through correction tests which imply the mixing with the normal plasma (1:1) = the normalization of the test means the presence of a deficit of the coagulation factors, while the persistence of their abnormality suggests the presence of a plasmatic inhibitor of the coagulation factors). 

 - Von Willebrand disease

 - The treatment with unfractionated heparin 

 - Direct inhibitors of thrombin 

 aPTT = N / PT ↑

 - Factor VII deficit 

 - Inhibitors of factor VII 

 - Vitamin K deficit 

 - Liver failure 

 - Treatment with cumarinic substances 

 a PTT / PT ↑

 - Factors II, V, X deficits and fibrinogen 

 - Inhibitors of factors II, V, X and fibrinogen 

 - Supratherapeutic doses of heparin or cumarinic substances 

 - Liver failure 

 - CID

 - Argatroban treatment

The drugs administered to the patient in the past and in the present can affect the hemostasis; the anamnesis must be systematically oriented towards this aspect too. Moreover, the anamnesis regarding the organic affections which can affect the hemostasis (liver diseases, liver and renal neoplasms, infections, etc.) must be done. 

 A last aspect which can favor important information for the diagnosis is the family history of hemorrhagic diathesis; this way we are able to answer the question whether the hemorrhagic syndrome is constitutional (hereditary, familial) or acquired. 

The confirming and analytical laboratory tests 

 These tests lead to the final diagnosis of the hemorrhagic disease and are generally the attribute of the specialized laboratories, which need technology and clever techniques, specialized laboratory personnel and are expensive. Their implications are obvious in cases of diagnostic difficulties, as in research, due to not being widely used in the usual clinical laboratories. These investigations also need specialized algorithms both in the execution and in their interpretation. 

Von Willebrand disease

 The structure and the functions of von Willebrand factor 

 VWF is an adhesive glycoprotein, present in the plasma in a concentration of 5-10 mcg/ml. A part of the VWF molecules circulate in the plasma complexed by f.VIII; a high active form is stored in the secretory granules of the platelets and in the endothelial cells. The moment these cells are activated by the tissue lesion (ex. by contact with thrombin), they instantaneously mobilize the stored VWF. 

 It is a big-size protein made up of many subunits, connected covalently by disulphide bonds. It contains numerous bonding sites for the platelets and the basal membrane of the blood vessels. It is made up of 2813 amino acids, having a molecular weight of D = 309.000; it also contains N – places and 0 – glycosylated. A propeptide of great dimensions (741 amino acids) is attached to the mature VWF. The propeptide is identical to AgVW FII and plays an important role in the assembly and processing process of VWF multimers. 

 The great forms of VWF assembled in endothelial cells are processed in the plasma in forms of little dimensions by the action of a plasmatic metalloproteinase, ADAMTS 13.

 In the plasma, VWF exists in the form of a series of multimers with the molecular weight which varies between 0,5 (dimers) and 20.000.000 D (multimers of great dimensions). From an electronomicroscopic point of view, it has a filament form and demonstrates the presence of many subunits arranged in a "head-to-head" and "tail-to-head" configuration. 

 The multimers construction block is the dimer, connected to other similar subunits by disulphide bonds situated near the N - terminal end of the mature subunit. 

 Disulphide bonds localized near the carboxy terminal end connect the multimers. 

 The mature subunit has a molecular weight of approx. 270 KD and contains 18,7% hydrocarbons. The hydrocarbons that are N – and – O connected are grouped at both ends of the subunits. 

 All the cysteine rests (169, representing 8,2% of the amino acids content of the molecule) are involved in sulphide bonds intra- and interbonds. 

 Two arginine-glycine-asparagine sequences (RGD) are present in Willebrand prefactor; one of them – also present in the mature subunit of VWF – takes part in the construction of the connection sites for GPII b/ III a platelet receptor. 

VWF protein demonstrates the presence of some repeated homologous segments, designated from H to D. A superfamily of proteins with a similarity of sequence with A domains of VWF contains proteins associated with the extracellular matrix, cellular adhesion and hemostasis. The crystalline structure of A1 and A3 domains of VWF is remarkably similar to the structure of the Ist domain of α2 / β1 integrin. 

 VWF structure is presented in Fig. 2. 

**Fig. 2:**
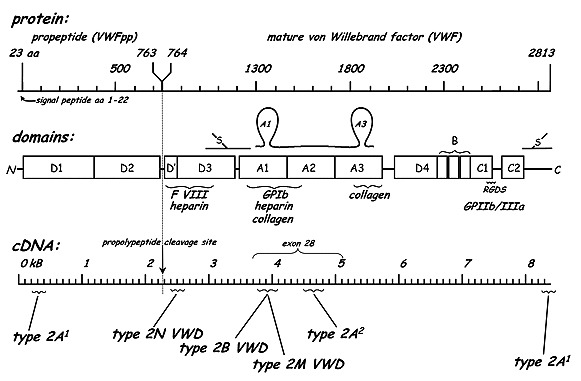
VWF structure

Many functional domains have been identified in VWF structure (**Fig. 2**): 

 - major places, physiologically active, of bonding the fibrillar collagen situated in H3 domain; 

- At least two heparin bonding places which can interact with the heparin-like molecules in the structure of the basal membrane of the blood vessels; 

 - the bonding domain of factor VIII situated in N-terminal side of the subunit which interacts with the N-terminal side of the light chain of molecule f.VIII; 

 - the bonding domain of platelet GPIb, situated at the level of a large disulfide bridge between the repetitions in H1 side of VWF molecule; 

 - the bonding domain of GPIIb/ IIIa on the activated platelets, situated in the carboxy terminal side of the mature subunit; 

 - the cleavage site for ADAMTS 13 at the level of H2 domain of VWF molecule. The biologic functions of VWF are the following: 

 - mediate the platelet adhesion (the attachment of the platelets at the level of the vascular lesions). VWF released from the endothelial cells from the vascular lesion are bonded with platelet GPIb and the collagen of basal vascular membrane, forming a bridge which resists the mechanic stress represented by the vascular flux; VWF is necessary in the initial attachment of the platelets at the level of the vascular lesion); 

 - it is involved in the platelet aggregation. Together with other adhesion platelets, such as fibrinogen, fibronectin and thrombospondin – VWF interacts with GPIIb/ IIIa on the activated platelets contributing to the platelet aggregation (the secondary aggregation induced by agonists); 

 - in VWF – factor VIII complex, factor VIII circular stabilizes, preventing its enzymatic destruction; in the absence of VWF, plasmatic T/2 of circulating f.VIII is more diminished, which proves the implication of VWF in coagulation. 

VWF gene 

 VWF gene is situated, in humans, on the short arm of 12/12p chromosome). It is a large dimensions gene (178 Kb, 52 exons, representing approximately 0,1% of the DNA of chromosome 12). 

 There is also a non-functional, partial duplication of the gene (VWF pseudo gene) situated on chromosome 22. It represents the duplication of the middle side of VWF gene, from the exons 23 to 34, including the intervening sequences. It has a homology of approximately 97.2% of the authentic gene and is only present in humans. 

 The structure of VWF gene is presented in Fig. 3. 

**Fig. 3:**
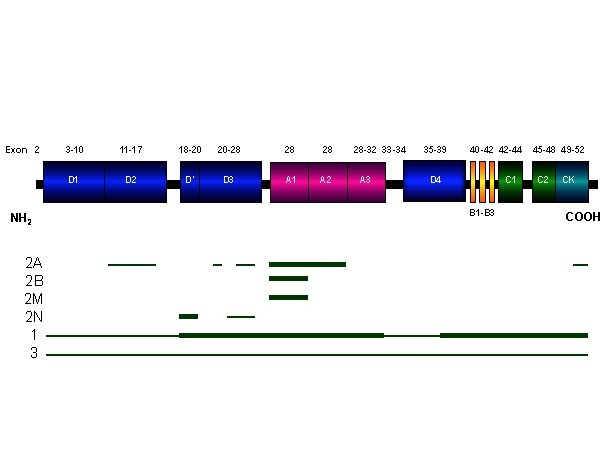
The structure of von Willebrand gene – specific mutations to different subtypes are shown in the image [3,5]

VWF biosynthesis 

 It is limited at the level of the endothelial cells and the megakaryocytes; VWF is initially synthesized as pre-VWF monomer. 

At the level of the endoplasmic reticulum of the endothelial cell, the transcription of the DNA takes place, producing a VWF RNA; the translation in RNA also takes place at this level. The dimmers are transported in Golgi apparatus. 

 At the level of Golgi apparatus, the N-linked hydrocarbonate is being processed, the O-linked glycosylation of the molecule and its sulfation. Moreover, the multimerization through disulphide bridges under the disulphide-isomerase, in acid environment is also initiated. 

The Post-Golgi multimerization (favored by the presence of propeptide) and the propeptide cleavage is continued. 

VWF storage and secretion 

 The storage is represented by the endothelial pool, at the level of Weibel-Palade corpuses (together with angiopoietin 2, tPA and IL-8), whose origin is in Golgi apparatus and the platelet pool, at the level of α granules. In both situations, the stored molecules form tubular structures, electromicroscopically evidenced and having a diameter of 150 Å. 

 A transmembranar protein – P selectin – is a component of α granules membrane and the Weibel-Palade corpuses. Its biosynthesis is restricted (just like the case of VWF) to endothelial cells and megakaryocytes. 

 The N-terminal domains (D1 – D3) are necessary for the storage of VWF; The selection of these domains leads to the constitutive secretion of VWF. 

 The secretagels are: thrombin, fibrin, histamine, C5 – C9 components of the complement, more bioactive lipids, for example sfingosine-1-phosphate and DDAVP for the endothelial cells. 

The molecular genetics of hereditary VWD 

 There are two types of pathogenic VWD: 

 - the quantitative deficit of VWF; it is realized through the deletion of a gene segment, or non-sense units which lead to the finishing of the synthesis of VWF protein by the insertion of a code stop in the coding sequence of VWF gene. 

 - qualitative structural or functional anomalies of VWF, generally produced through punctual mutations/ the substitution of only one nucleotide in the coding sequence and, so, of only one aminoacid at the level of VWF protein. 

The quantitative deficit of VWF can be severe or light. 

 In the severe quantitative deficit of VWF (type 3 of VWF), VWF is undetectable or has very low vales in plasma or platelets. 

 In light quantitative deficit (type I von Willebrand disease), there are low circular levels of VWF. The responsible mutations have been identified in approximately 55% of the studied cases. 

VWD history 

In 1926, Erik Von Willebrand described a 5-year-old Finnish girl, from Aaland Islands, with a hemorrhagic diathesis which was different from hemophilia A through the AR transmission, the presence of the cutaneous hemorrhages (and not joint-related) and the prolonged bleeding time [**1**]. 

The laboratory examinations in hereditary VWD subtypes

The researches in the last years have demonstrated that different mutations present in hereditary VWD have similar effects on the structure and function of VWF, which has led to a simpler classification of the disease, based on the pathogenic mechanism (**Table 4**). 

**Table 4:**
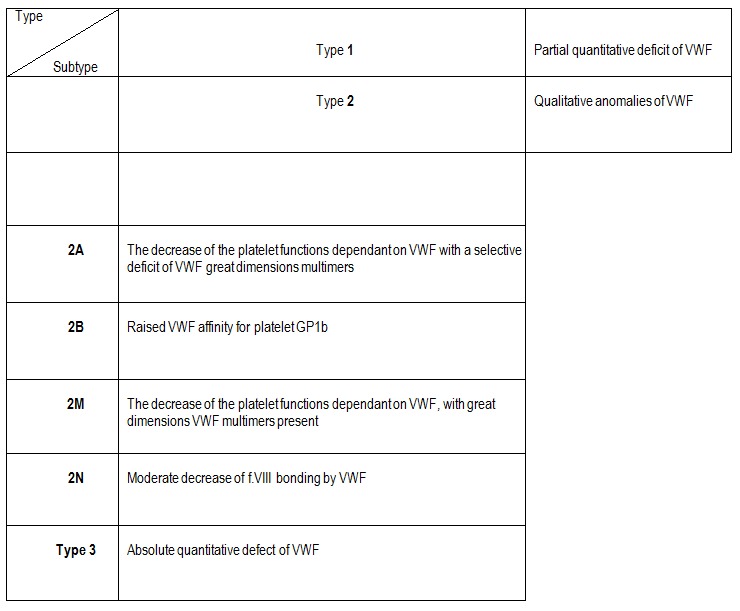
The pathogenic classification of VWD subtypes [**2**]

**Table 5:**
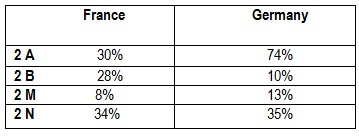
The repartition of 2 VWD subgroup in the population in Germany and France [**1**]

Table 6. Represents a synthesis of the main clinical and paraclinical characteristics of hereditary VWD subtypes [**2**]. 

**Table 6:**
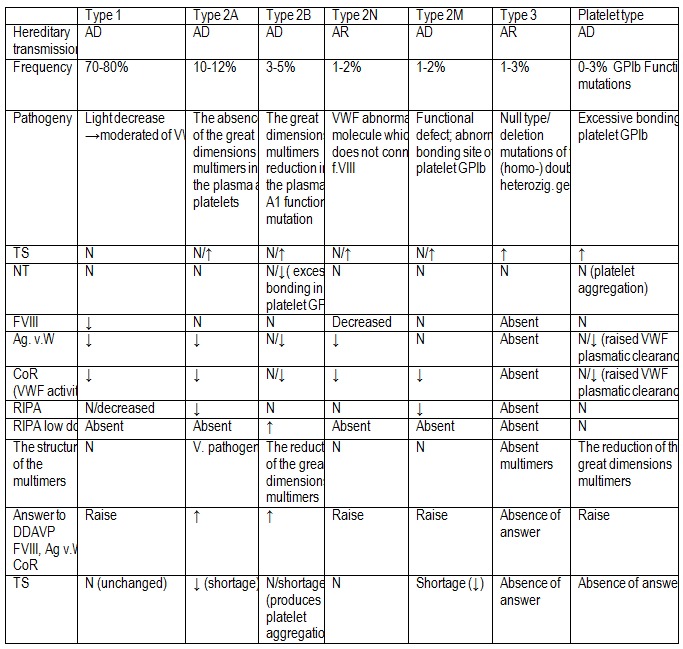
Subtypes of VWD
